# Saliva as a diagnostic fluid. Literature review

**DOI:** 10.4317/jced.50865

**Published:** 2012-10-01

**Authors:** Silvia Martí-Álamo, Aisha Mancheño-Franch, Cristina Marzal-Gamarra, Laura Carlos-Fabuel

**Affiliations:** 1 Degree in Odontology. Master in medicine and oral surgery. Dentistry department. University of Valencia

## Abstract

There is a growing interest in diagnosis based on the analysis of saliva. This is a simple, non-invasive method of obtaining oral samples which is safe for both the health worker and the patient, not to mention allowing for simple and cost-efficient storage. The majority of studies use general saliva samples in their entirety, complex fluids containing both local and systemic sources and whose composition corresponds to that of the blood. General saliva contains a considerable amount of desquamated epithelial cells, microorganisms and remnants of food and drink; it is essential to cleanse and refine the saliva samples to remove any external elements. Immediate processing of the sample is recommended in order to avoid decomposition, where this is not possible, the sample may be stored at -80ºC. Salivary analysis – much the same as blood analysis – aims to identify diverse medication or indications of certain diseases while providing a relatively simple tool for both early diagnosis and monitoring various irregularities. The practicalities of salivary analysis have been studied in fields such as: viral and bacterial infections, autoimmune diseases (like Sjögren’s syndrome and cɶliac disease), endocrinopathies (such as Cushing’s syndrome), oncology (early diagnosis of breast, lung and stomach carcinoma and oral squamous cell carcinoma), stress assessment, medication detection and forensic science among others. It is hoped that salivary analysis, with the help of current technological advances, will be valued much more highly in the near future. There still remain contradictory results with respect to analytic markers, which is why further studies into wider-ranging samples are fundamental to prove its viability.

** Key words:**Saliva, biomarkers, early diagnosis.

## Introduction

There is a growing interest in diagnoses based on salivary analysis given that collecting a sample of saliva is a simple, noninvasive method. Collecting oral samples is safe for both the health worker and the patient, not to mention allowing for simple and cost-efficient storage. These characteristics make it possible to monitor various biomarkers in children, the elderly and for those patients who do not collaborate in taking blood or urine samples. Another reason as to why saliva is a useful diagnostic tool is that there is a direct relation between the basic biochemical parameters in both saliva and blood (1).

Collecting saliva and sample handling

There are two important aspects to take into account when taking a saliva sample which could influence the results: the type of saliva – whole or gland-specific saliva -, and the level of stimulation – stimulated or nonstimulated saliva – (2).

In terms of this first aspect, the majority of studies (3-6) use general saliva; complex fluids containing both local and systemic sources which could be utilised for the diagnosis of oral pathology, salivary gland pathology and systematic diseases. Whole saliva is a complex fluid drawn from each of the major and minor salivary glands and the mucosal and periodontal fibre and which is directly affected by oral factors such as oral health problems. Collecting whole saliva samples is simple and requires minimal equipment (7). Other authors (8) prefer to collect saliva from one individual gland which, although being more difficult to collect and requiring more sophisticated equipment, it provides us with a more stable substance allowing for the retrieval of detailed information about diseases in specific glands thanks to the samples being less affected by the rest of the oral cavity (2). However, there are also authors who prefer to take samples of both types of saliva (9).

Whether the saliva is stimulated or nonstimulated is reflected in variable nature of the proportion of saliva in the major salivary glands and the concentration of certain proteins, ions and water. Nonstimulated saliva is considered to represent a neutral sample, less-affected by salivary glands, however it has also been suggested that stimulated saliva can allow for a more precise detection of cancer biomarkers. In cases of reduced salivary flow, like with Sjögren’s syndrome or after radiation therapy, salivary stimulation may be required in order to obtain an optimum amount of saliva (2).

The fact that saliva may become altered due to physiological processes which take place throughout the day and various oral stimulations must also be taken into account. In the majority of studies which have been carried out (3,6,8,9) participants have been requested to avoid oral stimulate such as eating, drinking and oral hygiene practises during a certain period before a sample is taken therefore establishing standard sample conditions due to the presence of circadian rhythms in saliva and their effect on the composition.

Given that general saliva contains a considerable amount of desquamated epithelial cells, microorganisms and remnants of food and drink; it is essential to cleanse and refine the saliva samples to remove any external elements. Immediate processing of the sample is recommended in order to avoid decomposition, where this is not possible, the sample may be stored at -80ºC (2).

## Using saliva as a diagnostic fluid

There is a large amount of literature which describes the use of saliva, as well as gingival crevicular fluid and oral mucosal transudates to both monitor medication and also detect various oral and systematic diseases. Salivary analysis, just like blood analysis, has two main objectives: to identify specific pathologies in patients and monitor change in those patients undergoing treatment (10).

The practicalities of salivary analysis have been studied in fields such as ( Table 1):

**Table 1:**
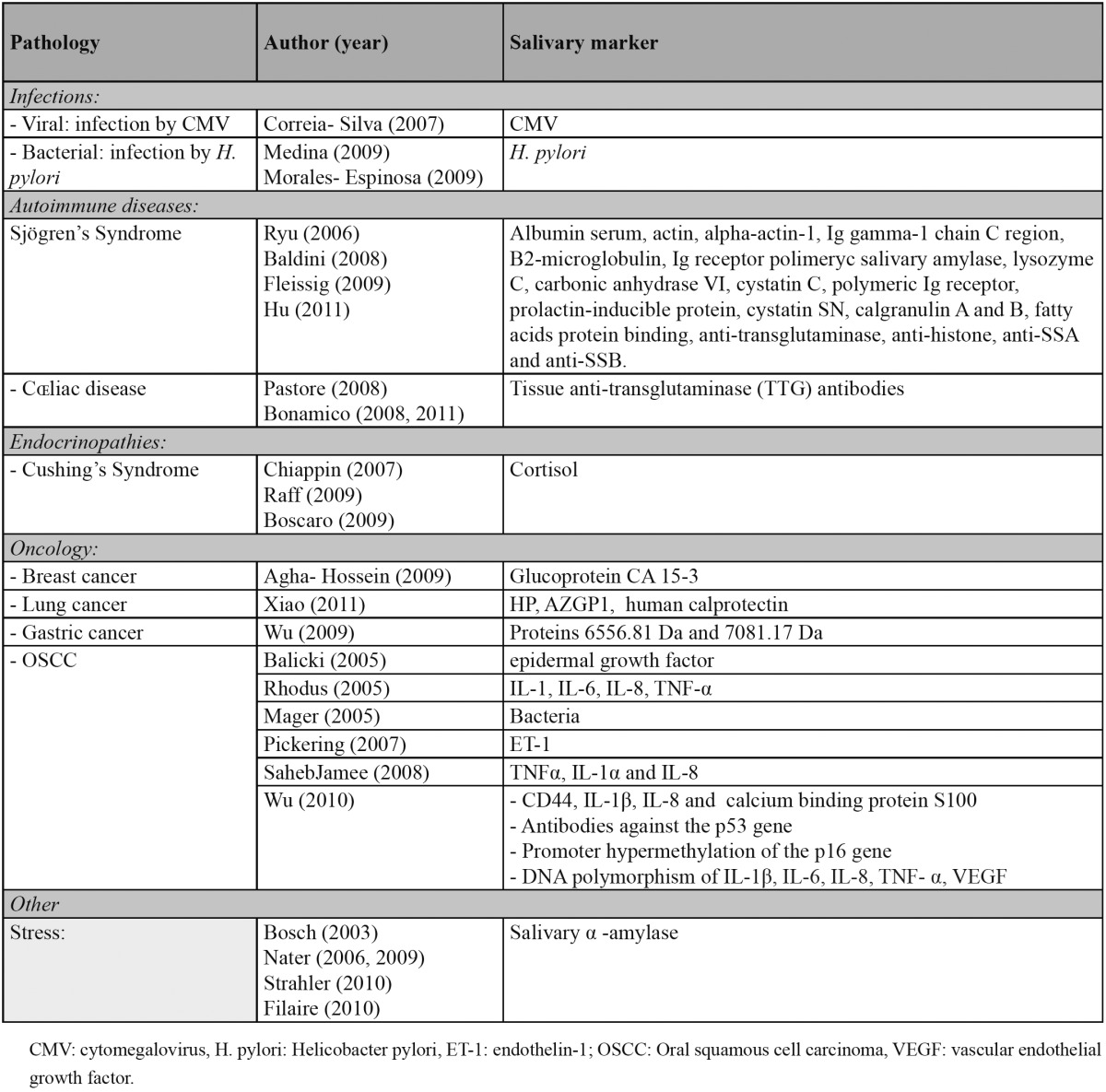
Diagnostic use of saliva.

1. Viral and bacterial infections.

2. Autoimmune diseases: Sjögren’s syndrome and cɶliac disease.

3. Endocrinopathies: Cushing’s syndrome.

4. Oncology: oral squamous cell carcinoma.

5. Cardiovascular diseases.

6. Medicinal and drug testing.

7. Forensic science.

1. Viral and bacterial infections

The Helicobacter pylori infection is related to a multitude of pathologies such as atrophic gastritis, gastric and duodenal ulcers and in some cases Mucosa Associated Lymphoid Tissue (MALT) lymphoma and gastric carcinomas. The oral cavity could become a reservoir for this type of pathogen, making a gastric reinfection possible (11,12).

The study carried out by Medina and cols. (11) proved that H. pylori was in fact present in oral samples (plaque and saliva) from patients with digestive pathologies whose results from gastric biopsies confirmed the presence of the bacteria. What’s more, the study confirmed that H. pylori was more commonly found in the oral cavities of patients who suffered from gingivitis and periodontal disease, which could be the reason behind resistance to the antibiotic treatment.

Morales-Espinosa and cols. (12) point out that the presence of H. pylori in the oral cavity is related to gastroesophageal disease and if accompanied by gastric symptoms, clear indications will be shown in the antibiotic treatment.

In 2007, Correia-Silvia y cols. (13) studied the presence of the cytomegalovirus (CMV) in saliva samples from 124 patients who had undergone a hematopoietic stem cell transplant. The presence of the virus was detected in all transplant patients whereas no trace of the virus was found in the specified healthy patients. The level of concentration of the virus was found to be at its highest 100 days after the transplant.

The authors reported that the state of immunosuppression in fact is likely to reactivate the virus, usually found within the oral mucosa, and that also the detection of CMV in saliva could be an extremely useful method for the early diagnosis of this infection.

2. Autoimmune diseases

Sjögren’s syndrome (SS)

Given that saliva is secreted directly from the salivary glands, analysis of salivary proteins in patients with SS is a promising area upon which to focus in the search of biomarkers for the disease. The analysis of proteins in saliva could also reveal biomarkers which differentiate primary and secondary variants of the syndrome (14). Biomarkers studied for the diagnosis of SS are numerous (2,3,8,9). Hu and cols. (15) have recently identified 24 antibodies which can differentiate patients with primary SS from patients with Systemic lupus erythematosus and healthy patients. In addition, they assessed four salivary antibodies (anti-transglutaminase, anti-histone, anti-SSA and anti-SSB in primary SS) in independent patients and they were successfully assessed using ELISA, displaying the potential of microarrays of highly active proteins in the detection of salivary antibodies acting as biomarkers. These biomarkers could become a clinical tool in the economic, non-invasive and simple detection of primary SS.

Cɶliac disease 

There are contradictory results concerning tests to detect the antibodies associated with the cɶliac disease (EC), and the definitive diagnosis continues to be based on an intestinal biopsy (16). However, according to certain studies, it might be possible to monitor the course of EC through the detection of tissue anti-transglutaminase (TTG) antibodies using the RBA (radio binding Assay) technique. Detecting these TTG antibodies could become a reliable, non-invasive easy procedure allowing us to monitor celiac patients on an adequate diet therefore avoiding the need for blood tests (4,5).

3. Endocrinopathies

Some hormones usually measured in plasma, such as steroids, non-steroids, peptides and protein hormones can be detected in saliva. Before determining clinical reasons for the various levels of hormones in saliva, it is important to define the correlation between levels of serum. Perhaps detecting steroidal hormones may be the most interesting focus of studies on salivary hormones. The most frequently utilised biomarkers are: cortisol, testosterone, dehydroepiandrosterone (DHEA), hydroxyprogesterone, progesterone and aldosterone (1).Cushing’s syndrome

Cushing’s syndrome is one of the most difficult endocrinopathies to diagnose, and for this reason researchers around the world have a keen interest in discovering a more efficient early diagnostic method (17,18). Diagnostic trials recommended by the Endocrine Society are: 24-hour urinary free cortisol, dexamethasone suppression test – 1mg oral at night and late-night salivary cortisol (17). Measuring cortisol in saliva is simple and reproducible and gives a reliable assessment of the hypothalamic-pituitary-adrenal axis (18). There are certain advantages with regards to cortisol serum tests, for example: the technique is simple, noninvasive and can be carried out in the patient’s home. For the reasons mentioned, the method is recommended in the treatment of children (1,17).

Salivary cortisol is in equilibrium with free cortisol in plasma and unaffected by the salivary flow. Following recent recommendations from the Endocrine Society, two late-night salivary cortisol measurements are encouraged in order to filter out those patients with a suspected hypercortisolism. It is also advised to measure salivary cortisol to study patients with cyclic Cushing’s syndrome and is it necessary to carry out a series of tests on paediatric patients.

Given all of the advantages mentioned, it is hoped that monitoring salivary cortisol will soon become a routine diagnostic method for Cushing’s syndrome.

4. Oncology

Validating markers for a variety of cancers such as breast cancer (19), lung cancer (20), gastric cancer (21) and oral squamous cell carcinoma (6,22-28) among others, is an extremely important tool for early diagnosis and in turn a higher survival rate for cancer patients.

The first salivary biomarker for cancer to be discovered was the HER2/neu for breast cancer (26). CA 15-3 is a prognostic marker for breast cancer given that levels appear higher when compared to the results of healthy patients. Agha-Hosseini and cols. (19) discovered a positive correlation between serum and salivary levels for CA 15-3 in an inactive salivary flow. They also observed higher levels of this marker in untreated and advanced breast cancer cases.

In 2011, Xiao and cols. (20) came across a statistically significant overexpression of the protein markers HP, AZGP1 and human calprotectin in patients with lung cancer. Similarly, Wu and cols. (21) came across higher levels of the 6556.81 Da and 7081.17 Da proteins in the saliva of a group of 23 patients with gastric cancer compared to healthy patients.

Oral Squamous Cell Carcinoma (OSCC)

The specific DNA and RNA of each tumour, just like the cells belonging to the lesion and inflammatory cells may be useful as markers of OSCC (26). Infections caused by microorganisms may also be implicated in the development of OSCC. Mager and cols. (28) detected an increase in the salivary expression of Capnocytophaga gingivalis, Prevotella melaninogenica and Streptococcus mitis in patients with OSCC compared with healthy patients.

An aberrant expression of CD44, IL-1β, IL-8 and calcium binding protein S100 has been detected in both protein and genomic techniques. Implementing genomic techniques we can observe promoter hypermethylation of the p16 gene in samples of serum and saliva. In addition, the DNA polymorphism of IL-1β, IL-6, IL-8, TNF-α and vascular endothelial growth factor (VEGF) has been associated with the development of OSCC. Autoantibodies against the p53 gene have been identified in samples of the saliva and serum of OSCC patients.

With the advances of nanotechnology applied to protein and genomic techniques, several markers of OSCC have been identified. However, more studies to confirm the specificity in larger sample sizes is most definitely required (26).

Along with OSCC patients, higher values of pro-inflammatory cytokines (IL-1, IL-6, IL-8, TNF-α) have been discovered in saliva samples from patients with premalignant lesions (with moderate or severe epithelial dysplasia) (27), and cytokine IL-8 in patients with periodontal disease and autoimmune diseases (6). However, these levels are not as high as those in patients with carcinoma (6,27). SahebJamee and cols. (25) did not find a significant statistical increase of TNFα, IL-1α nor IL-8 in the saliva of OSCC patients yet it must be taken into account that neither did they study a wide range of patients.

Balicki and cols. (22) observed a salivary increase in the epidermal growth factor (EGF) after head and neck cancer surgery which differed from the low levels of that marker before any cancer treatment took place. This is due to the fact that an over expression of EGF factors was detected in 75% of cancer patients. Pickering and cols. (24) uncovered a salivary concentration more than 3.5 times greater in patients with OSCC of the peptide endothelin-1 (ET-1). Both authors concluded that more studies are needed to determine the effects of EGF and ET-1 in the development of OSCC.

5. Stress

The salivary enzyme amylase α (AAs) has been suggested as a salivary marker which is sensitive to bodily changes in relation to stress (29,30).

The physiological response to stress consists of activating the sympathetic nervous system whereas the parasympathetic activity decreases. To assess this reaction, the direct determination of adrenaline and noradrenaline is extremely useful, for this reason AAs as a marker has been investigated (31). The AAs is an enzyme which hydrolyses glucose and maltose and which plays an important role in the oral phase of digestion aside from having an antibacterial function (30).

A wide range of studies (29,31-33) have shown an increase in both this enzyme and in sympathetic function as a direct reaction to psychosocially and psychologically stressful situations. Due to this type of stimulus, an increase has also been found in the secretion of salivary proteins such as MUC7 mucin, lactoferrin and general salivary proteins (32).

In short, depending on the stress stimuli, it has been proven that changes in this salivary enzyme are produced despite not yet understanding the biological meaning behind this phenomenon (30).

6. The detection of drugs and other medication

The use of saliva in drug and medication testing has grown and is replacing the traditional urine sample (34) ( Table 2). The concentration of drugs such as amphetamines, cocaine and some opiates in oral fluids are similar if not greater than in plasma (35) and in some cases drugs can appear in saliva before they appear in plasma (34).

**Table 2:**
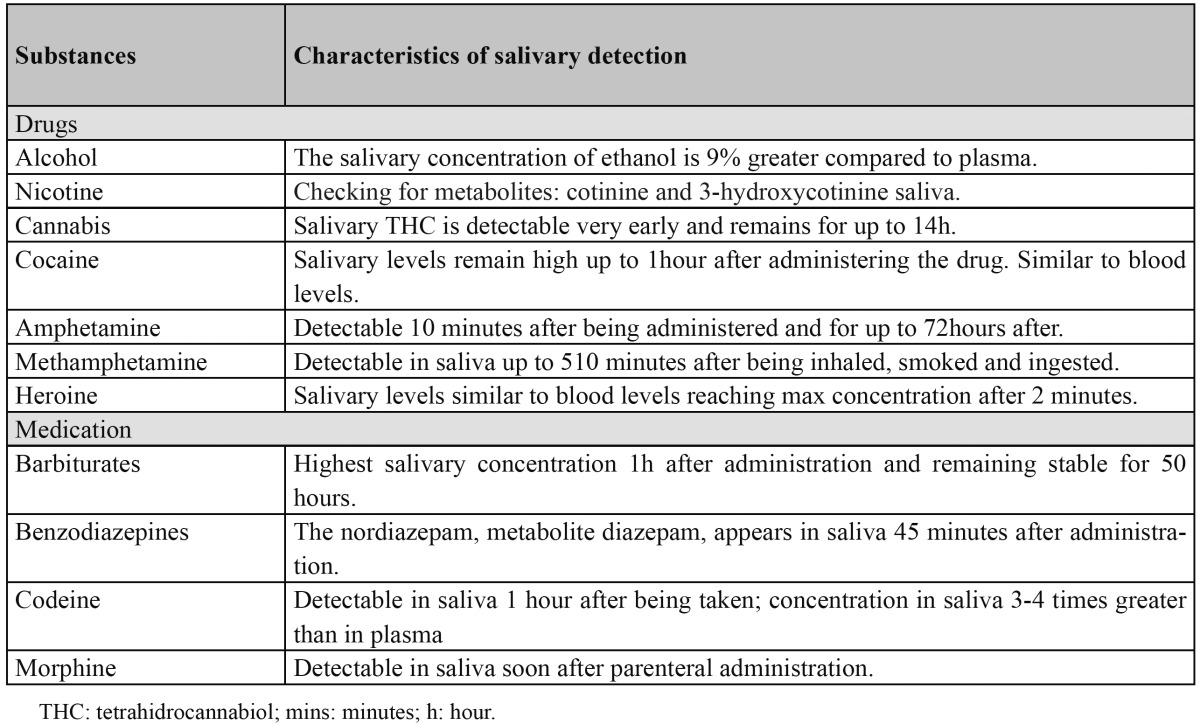
Salivary detection of drugs and other medication (34-36).

Alcohol is most commonly detected in toxicology analyses (35) especially in those connected to traffic accidents. The salivary concentration of ethanol is 9% greater compared to plasma (36).

When smoked, nicotine has a direct effect on the oral cavity which is why tests to determine exposure to this substance include analysing metabolites cotinine and 3-hydroxycotinine. The same effect is produced by cannabis which generally enters the organism by being smoked which explains why salivary levels are relatively high even without activating salivary flow (34).

Cocaine can be detected in oral fluid after any kind of administration, but more commonly after being smoked or inhaled. Salivary levels remain high up to an hour after administering the drug but decrease rapidly afterwards and are similar to levels found in the blood. Amphetamines are detectable ten minutes after being taken and last up to 72 hours whereas methamphetamines are detectable around 500 minutes after being inhaled, smoked or ingested (34,35). Levels of heroin in saliva are similar to levels in the blood and they are at a maximum just two minutes after being taken. These levels are greater if the heroin is smoked although around 30-60 minutes later they decrease reaching the values of peripheral blood (34).

There are few studies which analyse the presence of barbiturates and benzodiazepines in saliva due to their addictive nature. Higher concentrations of barbiturates have been found in saliva an hour after being administered remaining stable for 50 hours. In order to monitor the levels of benzodiazepines in saliva, the drug diazepam has been chosen because it tends to be more frequently used; its metabolite, nordiazepam, has been shown to appear in saliva about 45 minutes after administration (34).

Oral codeine will show up in saliva samples an hour after administration reaching a maximum concentration after 1.6-1.7 hours. The concentration of codeine in saliva is 3-4 times greater than in plasma (34,35). Morphine is detectable in saliva shortly after parenteral administration; however traces can also show after smoking heroin or eating poppy seeds (34).

7. Forensic science

Another interesting use of salivary analysis is in forensic science. Retrieving saliva, either directly from a suspect or from the injuries sustained by the victim, can facilitate the analysis of the DNA. In order to collect a saliva sample cotton swabs are used. Even dried saliva from bite wounds suffered by the victim can be analysed using an amylase assay kit (36).
